# Perceptions about hemodialysis and transplantation among African American adults with end-stage renal disease: inferences from focus groups

**DOI:** 10.1186/s12882-015-0045-1

**Published:** 2015-04-09

**Authors:** Megan L Salter, Komal Kumar, Andrew H Law, Natasha Gupta, Kathryn Marks, Kamna Balhara, Mara A McAdams-DeMarco, Laura A Taylor, Dorry L Segev

**Affiliations:** Center on Aging and Health, Johns Hopkins University, Baltimore, MD USA; Department of Epidemiology, Johns Hopkins School of Public Health, Baltimore, MD USA; Department of Surgery, Johns Hopkins University School of Medicine, Baltimore, MD USA; Johns Hopkins University School of Nursing, Baltimore, MD USA; Johns Hopkins Medical Institutions, 720 Rutland Ave, Turner 034, Baltimore, MD 21205 USA

**Keywords:** Dialysis, Kidney transplantation, Attitudes

## Abstract

**Background:**

Disparities in access to kidney transplantation (KT) remain inadequately understood and addressed. Detailed descriptions of patient attitudes may provide insight into mechanisms of disparity. The aims of this study were to explore perceptions of dialysis and KT among African American adults undergoing hemodialysis, with particular attention to age- and sex-specific concerns.

**Methods:**

Qualitative data on experiences with hemodialysis and views about KT were collected through four age- and sex-stratified (males <65, males ≥65, females <65, and females ≥65 years) focus group discussions with 36 African American adults recruited from seven urban dialysis centers in Baltimore, Maryland.

**Results:**

Four themes emerged from thematic content analysis: 1) current health and perceptions of dialysis, 2) support while undergoing dialysis, 3) interactions with medical professionals, and 4) concerns about KT. Females and older males tended to be more positive about dialysis experiences. Younger males expressed a lack of support from friends and family. All participants shared feelings of being treated poorly by medical professionals and lacking information about renal disease and treatment options. Common concerns about pursuing KT were increased medication burden, fear of surgery, fear of organ rejection, and older age (among older participants).

**Conclusions:**

These perceptions may contribute to disparities in access to KT, motivating granular studies based on the themes identified.

## Background

African Americans are overrepresented among those with end-stage renal disease (ESRD), comprising nearly one-third of ESRD patients [1], yet are significantly less likely to receive optimal care [2-5]. For instance, African Americans are less likely to be referred to a nephrologist prior to chronic dialysis initiation [6,7], putting this group at increased risk for non-arteriovenous fistula access at dialysis onset [8-10], hospitalization [11,12], morbidity, and mortality [13-16]. When referral does occur, it is more likely to happen during advanced stages of renal failure [7]. Not surprisingly, African Americans also suffer lower referral rates for kidney transplantation (KT) [17]. Certain sub-groups among African American patients may be even more likely to experience disparities in care. For example, females and older adults are underrepresented in access to transplantation (ATT) [18-23], which in part may be due to patient preferences, attitudes, and knowledge rather than purely clinical factors [24-26]. The influence of age and sex on treatment choice within an already disadvantaged group of African Americans, however, is poorly understood.

To better understand these disparities, in the context of poorly understood mechanisms, we used a qualitative approach. Qualitative research potentially leads to deeper insights into patient perspectives, experiences, and attitudes [27] —factors that influence clinical decision-making and treatment preferences. Qualitative approaches such as focus group discussions (FGDs) and in-depth interviews have been used successfully to explore reasons for non-adherence to immunosuppressive regimens [28], to identify patient difficulties in approaching live donors [29], and to elucidate patient concerns about high-risk organs [30]. Furthermore, results from qualitative studies have informed tailored educational interventions. For example, barriers to pursuing living donation identified using FGDs [29] were used to design a home-based educational intervention aimed at providing information about living donation to patients and their support system [30].

Because qualitative data may provide new insights into patient treatment choice and factors that contribute to disparities in ATT, which, in turn, may inform future quantitative studies and educational interventions, this study used a qualitative approach to gain new insight into age- and sex-related factors and concerns that might impact clinical decision-making among African Americans with ESRD. Specifically, we explored perceptions about dialysis and KT using FGDs, emphasizing depth and exploration, rather than quantification and generalizability, by seeking granular data from a relatively small sample of participants with personal experience of being treated for ESRD and undergoing hemodialysis [31].

## Methods

### Study design, participant recruitment, and participant characteristics

Between October 2010 and February 2011, 36 African American adults (11 men aged <65, 9 women aged <65, 11 men aged ≥65, and 5 women aged ≥65), drawn from seven for-profit hemodialysis centers in Baltimore, Maryland and the surrounding area, participated in FGDs about KT. Participants were approached by study staff who visited dialysis centers over several days to ensure that patients at every dialysis shift were approached. Eligibility criteria included undergoing hemodialysis for at least 6 months, English-speaking, able to participate in a FGD, and not listed to receive a kidney transplant. Recruiters approached each patient at all three shifts at each dialysis center. Approximately 280 patients were approached, of whom 107 were eligible and interested in participating. Of those, 46 patients were available on the date and time of the FGD and 36 actually attended. Four facilitated FGDs [32,33], separated by age and sex (males <65, males ≥65, females <65, and females ≥65 years) [34,35], were conducted to identify common experiences on dialysis and patient-perceptions about KT. Participant demographics by group are shown in Table 1.

**Table 1:** **Participant characteristics stratified by sex and age**

	**Age <65**		**Age ≥65**	
	**Males** ^**b**^	**Females**	**Males**	**Females**
	**n = 11 (%)**	**n = 9 (%)**	**n = 11 (%)**	**n = 5 (%)**
Age (yrs), median [IQR]	58 [52, 58]	53 [48, 54]	72 [69, 75]	70 [66, 71]
Married	2 (25.0)	5 (63.0)	6 (60.0)	1 (25.0)
Currently unemployed	8 (100.0)	6 (85.7)	9 (90.0)	4 (100.0)
Household income < $30 K	5 (71.4)	4 (66.7)	8 (88.9)	3 (100.0)
Post-secondary education	4 (50.0)	2 (25.0)	4 (40.0)	2 (50.0)
Insurance Type^a^				
Medicare	6 (66.7)	6 (75.0)	9 (90.0)	5 (100.0)
Medicaid	2 (22.2)	3 (37.5)	2 (20.0)	1 (20.0)
Private	2 (22.2)	4 (50.0)	5 (50.0)	2 (40.0)
Dialysis time (yrs), median [IQR]	4.0 [2.0, 7.0]	2.3 [2.0, 5.0]	4.0 [3.0, 5.0]	3.0 [2.4, 5.0]
Previous transplant	1 (12.5)	2 (25.0)	0 (0.0)	0 (0.0)

^a^Some participants had multiple types of insurance.

^b^One participant in the group intended for males aged < 65 was 68 years old.

All cells depict number (%) unless otherwise indicated. Data on marital status, education, and previous transplant were not available for 6 participants; data on employment status was not available for 7 participants; data on household income was not available for 11 participants; data on insurance type was not available for 4 participants; and data on dialysis time was not available for 2 participants. Percents were calculated among those for which the data was available.

### Focus group conduct

Formative work for focus group script development included a review of the literature, expert opinion from a multidisciplinary advisory panel, pilot testing in mock focus groups, and further refinement based on pilot test results. FGDs were conducted at the Johns Hopkins Hospital with a moderators and assistant staff present. At each FGD, a moderator asked guided questions (Table 2) to encourage discussion about participants’ attitudes and concerns about dialysis and transplantation. Each session lasted approximately two hours. Because conversations were intended to be free flowing, not every participant answered each question. Following the FGD, participants were provided with a small meal and a gift card. All participants provided written informed consent, and the procedures and script were approved by the Johns Hopkins University Institutional Review Board.

**Table 2:** **Questions from the focus group discussion script**

1)	Are you ever worried about your health?
2)	Do you discuss your health with anyone?
3)	How healthy do you consider yourself to be right now?
4)	On the piece of paper in front of you, I would like you to write three words that first come to mind when you think of getting a kidney transplant?
5)	What are some reasons you think that people don’t get a transplant?
6)	What are the benefits of getting a kidney transplant?
7)	What are the disadvantages of getting a kidney transplant?
8)	Do you think gender would affect a person’s pursuit for a kidney transplant?
9)	Do you think race, a black or a white person, would affect a person’s desire for a kidney transplant?
10)	Do you think age would affect a person’s desire for a kidney transplant?
11)	Do you have any concerns about transplant surgery? What are they?
12)	Has your nephrologists talked to you about getting a transplant enough?

### Data analysis

All FGDs were audio recorded, professionally transcribed verbatim, and coded for thematic content analysis in order to inductively derive concepts and core themes from the data. Two investigators independently coded the transcripts by hand, identified major themes, and then discussed any discrepancies or disagreements that resulted with a third investigator who adjudicated differences. After adjudication, comparisons across the groups were made. Representative quotations were selected to illustrate findings.

## Results

Four themes emerged: 1) current health and perceptions of dialysis, 2) support while undergoing dialysis, 3) interactions with medical professionals, and 4) concerns about KT (Table 3).

**Table 3:** **Themes and sub-themes identified according to focus group characteristics**

**Themes**	**Sub-themes**			
	***Males, <65***	***Females, <65***	***Males, ≥65***	***Females, ≥65***
Current health and perceptions of dialysis	unhealthy because of dialysis; mental & physical burden	healthy except for dialysis	healthy except for dialysis; thankful/fortunate	healthy except for dialysis; independent; thankful/fortunate
Support while on hemodialysis	unsupported by friends & family; supportive of/from fellow patients; desire to appear normal	limited support from friends/family; supportive of/from fellow patients	support from friends/family; supportive of/from fellow patients	support from friends/ family; supportive of/from fellow patients
Interactions with Medical Professionals	inadequate/uninformative; control-seeking; self-education	inadequate/uninformative; control-seeking; self-education	inadequate/uninformative; control-seeking; self-education	inadequate/uninformative; control-seeking; self-education
Concerns about KT	lack of knowledge; pill burden; fear of surgery; fear for donors	lack of knowledge; pill burden; fear of surgery; fear for donors	lack of knowledge; pill burden; fear of surgery; fear for donors; older age	lack of knowledge; pill burden; fear of surgery; fear for donors; older age

### Theme 1: current health and perceptions of dialysis

With the exception of requiring dialysis for ESRD and the exception of a few participants who mentioned comorbidities (e.g. diabetes, hypertension, obesity, and depression), many participants felt healthy because they engaged in many normal activities:

*I’m [healthy] besides dialysis. I’m able to walk home. If I’ve got something to do, I can keep pushing and get it done. —Male, age 63*

Moreover, despite their initial reluctance to start dialysis, and the cramping, changes in complexion, and scars that resulted, many participants (especially females) felt grateful for dialysis because it initially saved their lives:

*I’m thankful…I’m alive today because of [dialysis]. —Female, age 70*

Although acknowledging that dialysis restricted some activities, many females and older males tried to maintain a positive outlook on living with dialysis:

*Overall, I consider myself lucky…I know I’m not 100% healthy, but I still do my regular things. I just take it easy. —Female, age 47*

*I’m not going to let this get in the way of all the things that I have done before. I’m going to live, and I’m going to get back to normal as quickly as possible. —Male, age 68*

The older women additionally emphasized their ability to maintain their independence while on dialysis:

*When I get off dialysis, I get the regular bus, and I go shopping, or I’ll go to the movies, or go to whatever. The last place I’m going to go is home and get in bed. —Female, age 69*

Five older women mentioned traveling, and several women described pushing themselves to regain independence post-dialysis:

*I do all my shopping, keep my house clean…I do my own cooking, my own washing. I have to…You do that or stay in a nursing home. At first I was sitting at home. I took my time and got myself together because I was in a wheelchair and on oxygen. I graduated from the wheelchair to a walker, and from the walker to a cane. One morning I just woke up, I had the cane on my arm, I opened the door, I looked out, and I threw the cane. —Female, age 73, who cares for her 81-year-old husband who has been diagnosed with dementia*

*I said, ‘I ain’t seen the baby, and I’m in this nursing home. I’m getting out of here.’ I kept pushing myself till I got out of that nursing home. I was on oxygen and everything. —Female, age 69, motivated by the birth of a grandchild to leave her nursing home*

Like these older women, one older man was *determined after dialysis started to go back to normal as quickly as possible. —Male, age 68*

However, the remaining older men did not emphasize their efforts toward independence.

Younger males also acknowledged that dialysis saved their lives. In contrast to females and older adults, however, many younger males had a less positive outlook on life with dialysis, focused on the mental and physical toll of dialysis, and felt unhealthy because they were no longer able to work or travel:

*I used to feel like Superman. I’m unhealthy. This dialysis thing is like kryptonite. It sucked everything out of me. —Male, age 46*

Others described how the number of pills, cramping, sore arms, bad skin, painful joints, and poor sleep wore them down. Only one younger male expressed sentiments similar to those expressed by many of the females and older adults:

*I have very good days. I’m blessed with my dialysis. I don’t feel bad. I get up and do what I want to do basically, [but] not everything like I used to. —Male, age 63*

Although younger males tended to feel more negative about dialysis, almost all participants commented that dialysis rescued them from kidney failure and enabled them to continue living, comparing dialysis to death rather than comparing dialysis to other possible options:

*If I were really healthy, I would not be on dialysis, [but] I am healthier on dialysis than I would be if I were not on dialysis. —Female, age 65*

### Theme 2: support while undergoing dialysis

Younger men described others’ indifference toward their dialysis. For example, friends and family had difficulty keeping track of dialysis schedules. One man instructed his friends,

*Don’t call me on Tuesday, Thursday or Saturday—don’t call me. Don’t even come by. Don’t do nothing —Male, age 58*

but they still called and came to his home. Likewise, younger men recounted others’ lack of understanding about the burdens of dialysis:

*They don’t realize that this is constantly on our minds every day. —Male, age 46*

Another man wanted to appear normal, but in doing so, his friends found his limitations difficult to understand:

*If you even try to explain to them, you end up kind of explaining to them over and over again…They seem like they’re not accepting for what you tell them. You’re the same person, because we try to make ourselves normal, just like them, and they think we’re normal to a certain point, and they try and make you do stuff that you really can’t do or don’t want to do. —Male, age 63*

Another man did not want to discuss his dialysis in order to appear normal:

*I only talk about it when somebody else brings it up. And I try to keep things normal as possible. You know, I treat them the same. I want them to treat me the same. Most of the time I feel okay, so I don’t talk about it if I can. But if they bring it up and talk about it too long I cut them off. —Male, age 53*

While many younger men felt that their friends and family expected them to continue their normal responsibilities and activities, one man felt that his friends no longer interacted with him because of dialysis:

*People now don’t call me up like they used to. —Male, age 58*

While younger women described slightly more support from family than the younger men, most also tended to have limited support. For example, one woman with a supportive spouse was still upset that her family treated her as if she were not undergoing hemodialysis:

*Monday, Wednesday, and Friday has been my schedule for the last ten years, and they ask me where I’ve been. I’m a little upset about it. I don’t really talk to them about it. And when I talk to them about me, before I can get out what I’m finished, they talk about them. So, I just cut it off. My husband is the main source [of support]” —Female, age 60*

Several women also described differences in their children’s level of support:

*Sometimes I get out of the car, and I can’t hardly move and walk; it seems like if it’s not about [my daughter] then she don’t want to help me. And it really hurts me. Now, my son, that boy has been my rock. He’s been there, because there was times, I wasn’t going to kill myself, but I just felt like I wanted to die. I was just tired and overwhelmed…But it really does hurt me when I ask her to do something, it’s like she’s angry at me for being sick. And it really hurts me. I think she doesn’t know how to deal with it. —Female, age 47*

Another woman, who felt supported by her daughter, granddaughter, husband, and church members still described difficulties with the support she received:

*I have an eleven-year-old daughter. She worries about me more than my 30-year-old daughter. I had to ask to take her to see a therapist. That’s how bad she worries about my health. It really bothers me. I had to tell her, ‘I’m gonna be here. I’m not going nowhere.’ That girl had a fit. My oldest daughter, she’ll call me, ‘Ma, I need…Can I get…’ She don’t call to say, ‘How do you feel?’ —Female, age 49*

In contrast to the younger adults, the older men stated they were able to talk about their dialysis with family and felt this helped them to cope:

*I talk to others. I like to talk. It makes me feel better. —Male, age 72*

*It’s good to talk to somebody else and just get it off your mind so that somebody else knows what you’re putting up with, because if some day you are slowing down—you drag a little bit—somebody else is going to understand why you’re slowing down—why you’re dragging. It’s easier when somebody else hears it. At least I have found that to be true. It’s not that they helped, but they heard what I thought about, and then I feel okay and I go on with it. —Male, age 68*

Like the older men, the older women felt they were able to discuss their dialysis:

*I talk to people about it to a degree. I’m a very quiet person…So, I still don’t really talk a lot about it. If I’m really not feeling well, I’ll say that. —Female, age 69*

Generally, the older women seemed to have adequate support:

*I talk to people because of who I am…and I have a good support system because of my church family. I worked professionally for years, and all of those people contact me. I have children. —Female, age 70, whose sister took off of work to take her to a seminar on transplantation*

Another woman who was already living with her sister also had two children offer her a place to live.

For some participants, fellow dialysis patients provided emotional support beyond what they were receiving from their friends and family. Many participants described how dialysis patients encouraged one another and formed close bonds:

*At dialysis or the access center, I meet people that have been on dialysis for seventeen years, and I feel so much better. —Female, age 53*

*Our unit is like a family. I think if any one of us had a situation, we could go to one of them and talk. —Female, age 47*

One participant volunteered at the clinic and talked to new patients to let them know, *what it’s about, and it’s all right. It’s okay. You’ve just got to keep a positive attitude. —Female, age 49*

Similarly another participant intentionally spoke to dialysis patients on their first day about the dialysis machine. He explained that because he shared his knowledge,

*The next thing I know they’ll be looking for me all the time to sit beside them, because they say, ‘This guy, he talks me through it’ —Male, age 52*

### Theme 3: interactions with medical professionals

Many participants, regardless of sex or age, felt that dialysis center technicians treated them poorly:

*[The technicians] don’t know how to work the machine well, and they’re stabbing me to death…they don’t know how we’re feeling. I can explain to them a hundred-thousand times that they’re hurting me, pulling me, pulling to fast. —Male, age 46*

*Most of [the technicians] are not treating the patients right. Some of the techs are not being trained the way they should be trained. They let you know they are not doing the patients any favor; they’re doing a service. They’re forgetting who [the patients] are.” —Male, age 80*

Participants also felt that the technicians did not explain procedures clearly even when asked:

*[The technicians] didn’t explain nothing to me. The only thing they give you is some Tylenol —Female, age 65*

Similarly, with the exception of a couple of participants who felt fortunate to have excellent doctors, most participants reported that their nephrologists did not answer questions and spent inadequate amounts of time with them:

*When my doctor comes by, I want to know what my creatinine level is and about these other tests…but they never tell you that. You’ve got to ask them. —Male, age 58*

*The nephrologists don’t like to see you come in…he don’t explain nothing to you. They do what they want to do. —Female, age 73*

*I asked the nephrologist to ‘hold on a second. If you don’t mind, would you hold on a second. I’ve got a few questions, and if you can’t answer my questions here, can you make an appointment so I can come to your office and talk to you?’ If you look away, they’re off to the next person. You can’t get good answers…You leave a conversation with more questions than you had when you started the conversation. We’re just getting so much misinformation, and we really need concrete—absolutely concrete—information. We need good people to talk to. —Male, age 68*

In response to what participants felt was sub-optimal care, several participants, especially younger participants and men, described intentionally taking charge of their healthcare:

*[The doctors] were overmedicating the hell out of me. I was taking 22 pills…my body started shutting down…I almost damn near died. So at that point, I got with the doctors and nurses. I wanted to know why the hell am I taking this pill, what’s it for…I’m the final say so about it since I’ve got to suffer. I almost died. He’s a professional, but hell, I’m the victim…What I did after they nearly killed me about twice dead on that machine, I learned that I’ll be watching my blood pressure…I say ‘you’ve got to stop pumping’…what I’m saying is you’ve got to pay attention…And when it comes down to sticking I say ‘No fishing!’ I hold that arm. I say, ‘You stick only one stick’. You’ve got to be in charge of your recovery—your whole dialysis. —Male, age 58*

Another participant also self-monitored while on dialysis after a severe cramping experience:

*I check [my fluid levels] when I go now. I tell when I’m getting ready to cramp. —Female, age 42*

Yet another participant was selective about his technician:

*I’ve got certain techs that I ask for because this is my arm…when I know you’re the best, I ask for you because this is my arm. I don’t want it to blow up. I don’t care who gets mad. —Male, age 52*

Finally a couple of participants tried to educate themselves about renal disease and dialysis:

*One thing about being on dialysis is being knowledgeable as to what’s going on with you and stay up with what’s going on with you as far your dry weight and how much they’re going to take off that day and things like that…you try not to let them take off no more than that, not unless you request it. —Male, age 69*

Male and female participants of all ages also indicated that medical professionals provided very little information about transplantation:

*The biggest problem is that not enough information is available, and available timely, so that people have a chance to think and digest and maybe talk about it with other people before they make those kinds of decisions regarding transplant. —Male, age 68*

*[Doctors] need to tell us the pros, the cons, the results, what could happen, and what couldn’t happen. —Female, age 49*

A couple of participants suggested that financial incentives motivated physicians to keep patients on dialysis and to withhold information about transplantation:

*What it comes down to is money. If you see the prices they charge for these [dialysis] treatments. —Male, age 68*

### Theme 4: concerns about KT

Despite an overall lack in their desired level of knowledge about KT, several participants expressed interest in transplantation:

*I’m willing to take that chance…I want to get back to living and being busy. —Male, age 53*

However, most participants provided reasons for their reluctance to pursue transplantation including concerns about the number of pills they would need to take following transplantation, fear of allograft failure or organ rejection, and current success with dialysis:

*My main reason for why I don’t want a transplant is I’m not good at taking medications. —Female, age 65*

*When you get a kidney, you don’t know if it’s going to work or not. That’s why I didn’t put in for one. —Male, age 69*

*I would rather live with dialysis than get another kidney. —Female who had previously lost an allograft, age 47*

*I’ve seen more people who had a transplant and the next thing I know, I heard that they had passed away…30-40 in one dialysis clinic…I’m probably going to do better just doing dialysis treatment. —Male, age 52*

*As long as I can take the medication that I’m taking and it keeps me going, as long as my blood is washed and clean…I just don’t have no intention of getting a transplant. —Female, age 65*

*I stayed for so long because my dialysis is working fine. —Male, age 49*

A few participants were also afraid of surgery:

*I ain’t ready to be cut. —Male, age 52*

Some of the older participants mentioned their age as a reason not to undergo transplantation:

*I’m not going to do it; I’ve lived long enough. —Female, age 70*

*At my age, I’m not going to take the chance…I’m too old. I’ve lived a good life. —Male whose children wanted to donate a kidney, age 68*

Other participants who had friends or family members willing to be live organ donors refused because of fears related to the wellbeing of the donor:

*Even though they make it sound as if the donor will be fine, I just don’t want to bother [my son]. —Female whose son offered to donate his kidney, age 53*

*I told [my son] ‘no’ because he’s my only son, and I never know when something might happen to the one kidney he has. —Female whose son offered to donate his kidney, age 47*

One woman who asked her daughters to consider donating realized that her daughter was afraid and stopped asking her because she *wouldn’t do anything to further her fear. —Female, age 65*

## Discussion

In this qualitative study, we explored age-stratified and sex-stratified patient perceptions of experiences on dialysis and preferences about KT to gain in-depth insight into potential mechanisms contributing to health disparities in clinical decision-making regarding renal replacement therapy (RRT) among African American adults. Four themes emerged: 1) current health and perceptions of dialysis, 2) support while undergoing dialysis, 3) interactions with medical professionals, and 4) concerns about KT.

Patients’ perceptions about how well they are maintaining their independence and daily activities while undergoing dialysis influenced their attitudes toward their health and potentially toward pursuing KT. Females and older males tended to be more positive about dialysis experiences, possibly because they had more support from family and friends. In contrast, younger males, who tended to lack support from friends and family, expressed more frustration about being on dialysis. All participants shared feelings of being treated poorly by medical professionals, lacking information about renal disease and treatment options including transplantation, and desiring more knowledge about these options. These negative experiences with medical professionals and lack of knowledge possibly contributed to participants expressing a number of concerns about pursuing KT, such as medication burden, fear of surgery, fear of organ rejection, and older age (among older participants only). Conversely, almost all participants were grateful to dialysis for initially saving their lives, possibly reflecting an almost loyalty to this treatment modality because it was there when they needed it, while KT is something they need to pursue. Furthermore, the bonds that patients form during dialysis seem to create a social, emotionally supportive "dialysis family" that they might fear losing if they left the dialysis center and endeavored on the unknown, seemingly lone pursuit of transplantation.

While nephrologists potentially play an important role in patient-centered decision-making [22,36], participants often felt they were solely in charge of their health interests, lacked important health information, and had unaddressed concerns about transplantation. This finding is consistent with previous work demonstrating that patients perceive they have a choice in RRT, but participation in that choice and knowledge to make that decision is limited [25,36-39]. Moreover, African Americans are more likely to report inadequate information from their primary nephrologist and distrust their physicians’ medical decisions [26], which participants in this study also expressed. While others have reported that nephrologists are more likely to provide supplementary KT information and encourage patients to pursue KT in other urban settings [40], participants in this study did not have a similar experience. Taking measures to improve doctor-patient relationships, delivery of health information, and counseling about treatment options for ESRD may increase patient satisfaction and interest in pursuing KT.

Despite an average time on hemodialysis of 4.4 years, many participants, especially females, were reluctant to pursue KT. This finding is concerning given the survival benefits [41-44] and improved quality of life following KT for appropriate candidates [45] and given that increased time on dialysis is associated with decreased graft and patient survival post KT [46,47]. Our finding that African American females and older adults were less interested in pursuing transplantation is consistent with two large quantitative studies from 1998 and 1999 demonstrating that older adults and females report less interest in KT compared to younger adults and males [25,26]. However, our study introduces a potential explanation for these findings as older adults and females in our study also seemed to be more satisfied with their level of activity while on dialysis, stating that they felt independent and still traveled, which perhaps contributed to their reluctance to pursue KT. Indeed other studies have noted that independence and travel were perceived benefits of transplantation, but did not explore whether men and women, or older and younger adults, perceived these benefits differently [40]. One potential explanation for the difference in perceptions between older and younger adults is that patients perceive their level of activity in comparison to peers of similar ages; in other words, younger patients are more aware of their limitations because they are less active than the typical younger adult, while older patients are less aware of their limitations because their peers are more likely to have limited activity because of other chronic diseases. These findings suggest that older adults undergoing dialysis may benefit from focused conversations about known risks and improvements in survival and quality of life specific to their age group following KT.

This study has several limitations. First conclusions from our data are limited in that we only collected data from African Americans in Baltimore, MD, so no comparisons by race could be made, and our results may be less generalizable to non-urban as well as non-African American populations. Second, qualitative methods preclude determining quantitative relationships between factors, as the distribution of views is intended to be illustrative rather than representative. However, focus groups highlight a range of views of the target population, making this approach useful in generating hypotheses about relationships, informing further research, and providing insight into social phenomena in the healthcare context [48]. Indeed, we have identified several ways in which males and females or older and younger adults differ in their perception of dialysis, which previously have not been identified and which possibly contribute to a greater reluctance among females and older adults to pursue KT. Third, self-reporting bias may occur during FGDs, but the method is effective in gleaning detailed information from a small number of participants. Finally, we chose to conduct the qualitative data analysis by hand. While software includes useful tools for viewing data and organizing matrices, the amount of data from the FGDs could be easily managed, organized, and searched by hand or by using search tools available in software not specific for qualitative analysis.

## Conclusions

In conclusion, this qualitative study suggests potential age and sex differences among African Americans in perceptions of experiences undergoing hemodialysis, loyalty to dialysis as a treatment modality, social support from their "dialysis family" and/or their own family, and perceived barriers to pursuing KT. This study also suggests a high level of inadequate knowledge about KT regardless of age or sex. The novel themes identified in this study may facilitate the development of further quantitative studies that assess differences in dialysis experiences, choices about RRT, and pursuit of KT by age and sex. For example, quantifying how sex or age might modify the association between perceived benefits or risk of transplantation might be useful in understanding mechanisms of disparities in access to KT. These granular findings also may help inform the development of educational programs that aid females and older adults in overcoming barriers to pursuing transplantation by emphasizing the benefits of transplant and better explaining risks for appropriate candidates, and in turn, reduce disparities in access to KT, specifically among African Americans who remain a particularly vulnerable group with renal disease. Finally, these results underscore the need for better patient-physician communication in regard to patient understanding of health status, current treatment, and treatment options, particularly among patients who may be more vulnerable to health disparities.

## Abbreviations

ATT: Access to transplantation
ESRD: End-stage renal disease
FGDs: Focus group discussions
KT: Kidney transplantation
RRT: Renal replacement therapy
